# A Novel Uni-Acupoint Electroacupuncture Stimulation Method for Pain Relief

**DOI:** 10.1093/ecam/nep104

**Published:** 2011-01-09

**Authors:** Chuansen Niu, Hongwei Hao, Jun Lu, Luming Li, Zhirong Han, Ya Tu

**Affiliations:** ^1^Department of Mechanical Engineering, Tsinghua University, Beijing, China; ^2^Institute of Man-Machine and Environmental Engineering, School of Aerospace, Tsinghua University, Beijing, 100084, China; ^3^School of Acupuncture, Beijing University of Chinese Medicine, Beijing, China

## Abstract

Electroacupuncture stimulation (EAS) has been demonstrated effective for pain relief and treating other various diseases. However, the conventional way of EAS, the bi-acupoint method, is not suitable for basis study of acupoint specificity. Moreover, its operations are inconvenient and difficult to be persevered, especially for long-term, continuous and even imperative treatments. These disadvantages motivate designs of new EAS methods. We present a novel uni-acupoint electrical stimulation method, which is applied at a single acupoint and quite meets the needs of basis study and simpler clinical application. Its pain relief effect has been evaluated by animal tests of Wistar rats. During the experiments, rats were given 30 min 2/100 Hz uni- and bi-acupoint EAS and their nociceptive thresholds before and after EAS were attained by hot-plate test. The analgesic effect was defined as the change of nociceptive threshold and used to evaluate the effectiveness of uni-acupoint EAS for pain relief. The hot-plate test results indicated that analgesic effect of uni-acupoint group was significantly higher than that of the control group and there was no significant difference of analgesic effects between uni- and bi-acupoint EAS. The results suggested that uni-acupoint method was an effective EAS method and had comparable pain relief effect with bi-acupoint method.

## 1. Introduction

Acupuncture has been practiced for thousands of years in China and other Oriental countries. It has recently been recognized in Western world and gradually accepted as a beneficial form of complementary medicine [1]. As a combination of acupuncture and electric current, electroacupuncture stimulation (EAS) was demonstrated as effective for pain relief in 1970s [2] and since then it has been widely used for treatment of various diseases [3–8]. EAS is a landmark development of traditional manual acupuncture, in which the stimulus applied to acupoints is no longer empirical manipulations but quantitized electric currents. It provides a replicable method for the scientific study of acupuncture. According to types of electrodes, EAS can be classified into needle EAS and transcutaneous EAS (TEAS), in which needles inserted into the acupoints and self-adhesive electrodes placed on the skin above the acupoints are used to deliver the stimulating current [9].

Acupoint specificity is an important basis of acupuncture in traditional Chinese medicine (TCM). According to the TCM theory as well as clinical practices, different acupoints play different roles and acupoints should be selected carefully for curing different diseases [10, 11]. Although the physiological mechanism still remains unclear, studies in animals have shown that EAS applied at certain acupoints can facilitate the release of neuropeptides in central nervous system (CNS) [9, 12] and studies in humans with functional magnetic resonance imaging [11, 13] or positron emission tomography [14] provided primary evidences of the existence of acupoint specificity, but more scientific investigations are needed. However, for almost all the existing research and clinical applications, EAS is implemented in bi-acupoint method, in which pairs of acupoints are selected to establish the stimulating current loop [5, 11, 15, 16]. When a bi-acupoint EAS is applied, stimulating current flows between two selected acupoints, and it activates acupoints and meridians distributed on the pass way of the current. Thus, the resulting therapeutic effect would be a combination result of those produced at various parts. Therefore, bi-acupoint EAS method cannot meet the requirements of acupoint specificity study. In addition, the placement and removal of electrodes required for each application of both needle EAS and TEAS are troublesome. So, bi-acupoint EAS, which has to use two electrodes for each channel of stimulating current, is inconvenient and difficult to be persevered, especially for long-term, continuous and even imperative treatments, such as severe chronic pain, depression and drug addiction.

To overcome these disadvantages, we present a novel uni-acupoint electrical stimulation method based on a single acupoint. In this article, we evaluated its efficacy of pain relief, one of the most typical functions of EAS, in Wistar rats by hot plate test.

## 2. Methods

### 2.1. Novel Uni-Acupoint Electrical Stimulation Method

**Figure 1:**
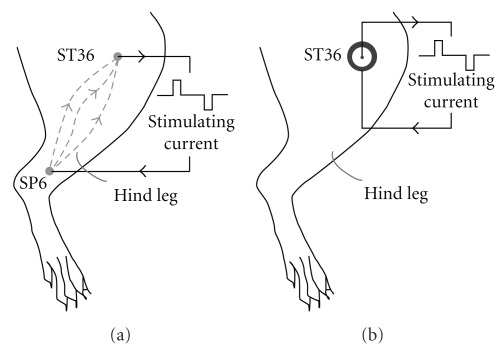
Comparison of bi-acupoint and uni-acupoint EAS methods. (a) Bi-acupoint EA stimulation applied via Zusanli (ST 36) and Sanyinjiao (SP 6) in rat left hind leg [12]. (b) Uni-acupoint EA stimulation applied via single acupoint, Zusanli (ST 36), in rat left hind leg. Zusanli is near the knee joint, 2-mm lateral to the anterior tubercle of the tibia and Sanyinjiao is near the ankle joint, at the level of the superior border of the medial malleolus between the posterior border of the tibia and the anterior border of the Achilles tendon.

**Figure 2:**
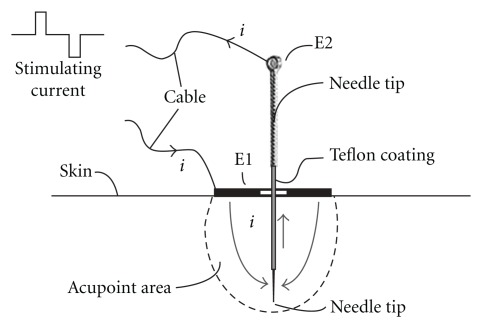
Diagrammatic sketch of uni-acupoint EAS method.

Bi-acupoint EAS method needs pairs of acupoints, e.g. Zusanli (ST 36) and Sanyinjiao (SP 6) in one leg [12, 17, 18], to form the current loop (Figure 1(a)), while uni-acupoint method can be applied on a single acupoint (Figure 1(b)) with two electrodes, E1 and E2 (Figure 2). E1 is a thin-ring made of stainless steel and used as a positive electrode in the experiment. The size of E1 electrode is 8 mm of external diameter, 2 mm of internal diameter and 0.05 mm of thickness. It is fixed on the surface of skin above the selected acupoint target using translucent medical adhesive tape, with the central hole aiming precisely at the selected acupoint. The conductive gel is applied on the shaved skin around acupoint to minimize the contact resistance between E1 and the skin. E2 is an ordinary acupuncture needle (Φ 0.25 × 13 mm) treated by isolation process. The body of E2 is covered by a thin layer of Teflon with the thickness of 50 *μ*m. The handle and 4-mm tip of the electrode are conductive. It is inserted into the acupoint through the central hole of E1 and used as negative electrode. To keep well-contact between E1 and skin as well as no shorting between E1 and E2, the resistance between E1 and E2 should be in the range of 1-2 kΩ, which is determined in previous experiments. When the stimulation is being proceeded, stimulating current flows into the acupoint area from E1 and out from the conductive tip of E2. As a result, the current will concentrate just in the selected acupoint area.

### 2.2. Animals

Adult female Wistar rats weighing 180–220 g were used in the experiments. They were housed collectively in rat cages (six per cage) under controlled environmental conditions (20–22°C) before experiment, with free access to food and water. All experimental procedures were approved by the University Committee on Research Practice at Beijing University of Chinese Medicine and the NIH guide for the care and use of laboratory animals [19] was strictly followed during the experiments.

### 2.3. Nociceptive Threshold Test

EAS-induced analgesic effect was evaluated by the change of nociceptive threshold (NT) measured in hot-plate test [20]. The hot-plate testing instrument (Chengdu TME Technology Company, China) used in our experiment mainly consisted of a glass container and a stainless steel floor. When the test was performed, the temperature of the stainless steel floor was set to 55 ± 1°C. The rat was put on the floor. When it could not endure the temperature of the floor, the rat would lick its hind paw. Then it was taken out of the glass container and put back to the rat cage. The time from the rat was put on the stainless-steel floor to the hind-paw licking was defined as NT.

### 2.4. Experimental Procedures

Forty-eight rats were divided in turn into four groups: control (*n* = 12), sham (*n* = 12), uni-acupoint (*n* = 12) and bi-acupoint (*n* = 12). The experiment assistants who actualized the rats grouping and NT measurements were blind to the study.

#### 2.4.1. Basal NT Measurement

Rats were taken out of rat cages and put into the hot-plate test instrument. The typical NT was 4–9 s. In order to avoid injury to rats, a maximum threshold of 20 s was set up in the tests. When the tests were finished, rats were taken back to rat cages. For each rat, its NT was measured three times with a 5-min interval and the average of three measurements was defined as the basal NT.

#### 2.4.2. EAS

**Figure 3:**
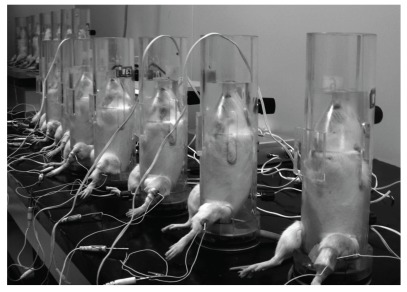
Rats receiving uni-acupoint EAS.

Rats were taken out of rat cages and kept in a special transparent Lucite barrel with tails and hind-legs left out naturally. These Lucite barrels (Shandong Medical Science Equipment Station, China) were designed to restrict rats and ensure the successful EA operations to these clear-headed rats. In order to minimize the bias effect induced by barrel restraint, rats were placed quietly for 15 min to be stabilized. Then, these rats were given different EA treatments in groups, as following:

 
*Control group:* No electrodes placement and no EAS.
 
*Sham group:* Electrodes placed but no EAS. Ring electrode was placed above left ST 36 and Teflon treated needle was inserted into ST 36.
 
*Uni-acupoint group:* Same electrodes placement as Sham group. Thirty-minute EAS (Figure 3).
 
*Bi-acupoint group:* Ordinary acupuncture needles (Φ 0.25 × 13 mm) were inserted into ST 36 and SP 6 of both hind legs. Two needles in the same hind leg were used as a pair to form the current loop [12]. Rats were given 30 min EAS with the same stimulating parameters as the uni-acupoint group.

We developed a four-channel programmable accurate constant current generator, TSC-V1, as the EAS stimulator. Bilateral symmetrical current pulse was used as the stimulating signal to zero the net electron charge flowing into the body [21]. The frequency of stimulating current was 2/100 Hz, 2 Hz alternated with 100 Hz and each lasting 3 s. The pulse width was 0.6 ms at 2 Hz, 0.2 ms at 100 Hz and the amplitudes changed from 1 to 2 mA and 3 mA every 10 min [9, 12]. The purpose of increasing intensity in steps was to allow the rats to gradually adapt to the EAS and to minimize the possible stress that might be induced by the higher intensity [9, 12]. No signs of suffering such as struggling or vocalization, except slight muscle tremors were observed during the stimulation.

#### 2.4.3. Post-EAS NT Measurement

After 30-min EAS was finished, the rat was released from the barrel and put into the hot-plate test instrument for the first post-EAS NT test, which was followed by other three identical NT measurements with the interval of 10 min.

### 2.5. Data Processing and Statistical Analysis

NT change at each test point was calculated as 100% × (post-EAS NT − basal NT)/basal NT [12]. The average NT change was defined as the average of NT changes at the end, 10, 20 and 30 min after the end of the stimulation. It was used to evaluate the overall analgesic effect of 30-min EAS.

The results were expressed as mean ± standard errors of the mean (SEM). The significance of statistical differences was determined using repeated-measures two-way analysis of variance (ANOVA) followed by least significant difference (LSD) *post hoc* test for NT changes, and one-way ANOVA followed by LSD *post hoc* test for average NT changes. *P*-values of <.05 were considered significant.

## 3. Results

### 3.1. Uni-Acupoint EAS Is Effective for Pain Relief

**Figure 4:**
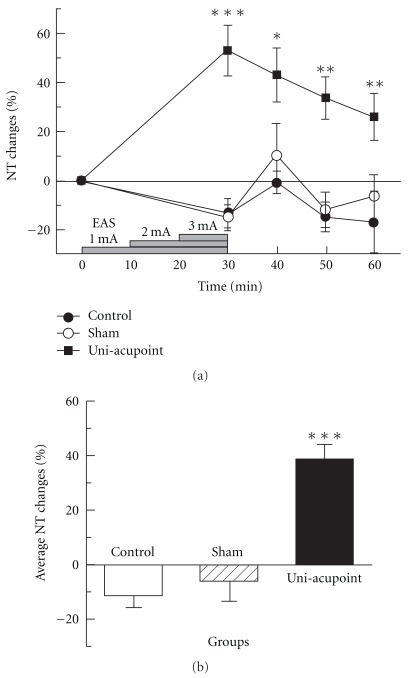
NT changes induced by EAS in control, sham and uni-acupoint EAS groups. (a) NT changes tested before and after EAS. (b) Average NT changes of different groups. *P < .05, **P < .01, ***P < .001, compared with control group.

As shown in Table 1, there was no significant difference of basal NTs between the four groups (*P* = .250). The NT changes of the control, sham and uni-acupoint groups are shown in Figure 4. For control group, NT changed indistinctively during the total 60-min experimental time and the average NT change was only -11.61 ± 4.17%. The slight change might be related to the effect of barrel restraint. Similar NT changes were observed in sham group and there was no significant difference compared with control group (*P* = .409). The results showed that operations including electrode placement and needle insertion of uni-acupoint EAS method would not induce distinguishable analgesic effect. For uni-acupoint group, NTs measured at four test points were all significantly higher than those of control group. NT reached its peak value at the end of EAS and decreased with time after that. The average NT change (38.82 ± 5.60%) in uni-acupoint group is significantly higher than the control group (*P* < .001). These results demonstrated that uni-acupoint EAS was effective for pain relief.

### 3.2. The Analgesic Effect Induced by Uni-Acupoint Is Comparable with That of Bi-Acupoint Method

**Figure 5:**
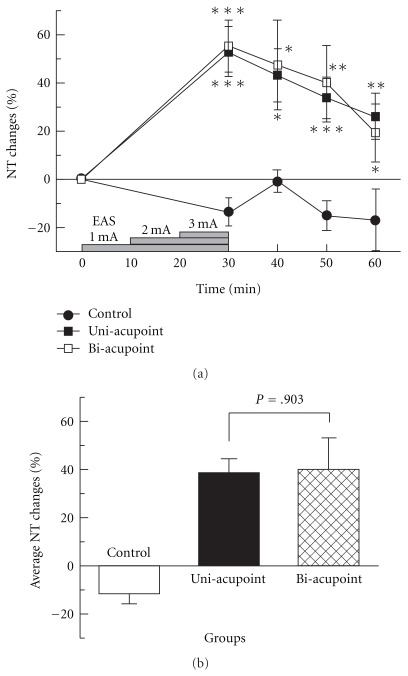
Comparison of NT changes induced by EAS in uni- and bi-acupoint EAS groups. (a) NT changes tested before and after EAS; (b) average NT changes of different groups. *P < .05, **P < .01, ***P < .001, compared with control group.

During total 60-min observation time, the trend of NT changes in uni-acupoint EAS group was similar to that in conventional bi-acupoint needle EAS applied at Zusanli and Sanyinjiao in both hind legs. There was no significant difference between the two groups (*P* = .852). Average NT change in uni-acupoint group was a slightly little lower than the bi-acupoint group (40.27 ± 12.71%), but there was no significant difference between them (*P* = .903) (Figure 5(b)). These results suggested that the analgesic effect induced by uni-acupoint was comparable with that of bi-acupoint method.

## 4. Discussion

### 4.1. Uni-Acupoint EAS Method for Pain Relief

Compared with manual acupuncture, EAS produces more clinically efficacious analgesia, and is also a more standardized stimulus for scientific study [22, 23]. Our work suggested that EAS can be applied via uni-acupoint method based on a single acupoint, in addition to conventional bi-acupoint method. Uni-acupoint method has been proved effective for pain relief, by the observed significant NT increases at the termination and during the following 30 min of EAS.

**Figure 6:**
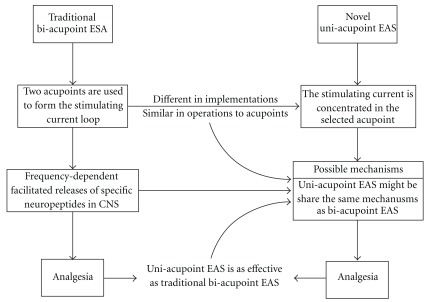
The hypothetical diagram for effectiveness validation and possible mechanisms investigation of uni-acupoint EAS method.

Although the clinical applications of bi-acupoint EAS have increased rapidly, the mechanism behind it still remains unclear. It has been reported that EAS-induced analgesic effect might be closely related with the frequency-dependent release of specific neuropeptides in CNS: the low (2 Hz) and high (100 Hz) frequency EAS facilitates the releases of endomorphin and dynorphin, respectively, and 2/100 Hz (stimulating current alternating between 2 Hz for 3 s and 100 Hz for 3 s) EAS promote maximal releases of opioid peptides and produce strongest analgesia [9, 12, 24]. It is known that for pain relief and other virtues of EAS, the responses in CNS are triggered by afferent impulses induced by peripheral electrical stimulation [9]. Although uni-acupoint and bi-acupoint EAS are very different in implementations, they are similar in operations to the acupoint. Therefore, we consider that uni-acupoint EAS might share the same possible mechanism with bi-acupoint EAS. The hypothetical diagram for effectiveness validation and possible mechanisms investigation of uni-acupoint EAS method is shown in Figure 6. The pain relief effect of uni-acupoint EAS can be improved by changing stimulation parameters, such as selecting optimal frequency as well as increasing the intensity, which has been proved effective for bi-acupoint EAS [9, 12, 25].

The differences between uni- and bi-acupoint methods, mainly including efficiency of stimulating signal and stimulating zones, might result in difference between their analgesic effects. First of all, the efficiencies of the bilateral symmetrical stimulating signal are different for the two EAS methods. When the stimulation is being applied, neuron membranes are depolarized as the positive charges flow outwards. As a result, action potential occurs and afferent impulses induced by electrical stimulation are transmitted to the CNS. On the other hand, when the positive charge flows in, neuron membranes is hyperpolarized [26] and no action potential will be triggered. Therefore, in bi-acupoint EAS, either positive or negative phase in each integrated simulating pulse will result in afferent impulses at one of the two acupoints in bi-acupoint EAS. These afferent impulses will merge in the reticular formation of the brainstem and facilitate the release of opioid peptides related to analgesia [9, 12]. However, in uni-acupoint EAS, only positive half is useful for analgesia, and the other half pulse mainly plays a role of charge balance. Secondly, the stimulating areas of two methods are different. As mentioned above, bi-acupoint EAS needs pairs of acupoints, for example, Zusanli/Sanyinjiao (ST 36/SP 6) to form the stimulating current that activates all the acupoints and meridians distributed between the two selected acupoints in bi-acupoint EAS and a mixed analgesic effect is gained. Differently, when flowing between the two electrodes in uni-acupoint EAS, the stimulating current is limited in the selected acupoint area and no other acupoints or meridians are influenced.

### 4.2. Uni-Acupoint EAS Method for Acupoint Basis Study and Clinical Application

Compared with bi-acupoint EAS, uni-acupoint EAS is much more like the traditional manual acupuncture, because it mainly concentrates its stimulating current in the selected acupoint area and induces no direct activation at other parts. Exactly for this reason, it is possible to confirm and compare the specific functions of single acupoint or acupoint groups with uni-acupoint method. As another result, the difference between electroacupuncture and ordinary electrical stimulation could be differentiated. Besides, because uni-acupoint is applied at a single acupoint, the two electrodes used for each channel of stimulating current could be integrated in one electrode pad. Therefore, the clinical operations of EAS will be much simplified by electrodes halving.

In summary, uni-acupoint EAS method applied based on a single acupoint is proved effective for pain relief. Due to its special design, uni-acupoint EAS will also have great potential for acupoint specificity study and simplifying routine clinical EAS operations.

## Funding

National High Technology R&D Program of China (863 program) (Grant no. 2006AA02Z4E9) and National Key Technology R&D Program of China (Grant no. 2006BAI03A18).

## Figures and Tables

**Table 1:** Basal NTs of four groups.

Basal NTs of different groups				*P*-value
Control	Sham	Uni-acupoint	Bi-acupoint	
6.53 ± 0.35	6.94 ± 0.28	6.15 ± 0.28	6.71 ± 0.32	.250
